# Autophagy-mediated TET2 degradation by ALV-J Env protein suppresses innate immune activation to promote viral replication

**DOI:** 10.1128/jvi.02003-24

**Published:** 2025-01-22

**Authors:** Shihao Chen, Jiaxing Wang, Qiangzhou Wang, Yuyu Ping, Ting Yang, Kejiao Ma, Shiyu Pan, Yulin Bi, Hao Bai, Guobin Chang

**Affiliations:** 1 College of Animal Science and Technology, Yangzhou University 614678 , Yangzhou, Jiangsu, China; 2 Joint International Research Laboratory of Agricultural & Agri-Product Safety, The Ministry of Education of China, Yangzhou University 614792 , Yangzhou, Jiangsu, China; 3 College of Veterinary Medicine, Yangzhou University 614704 , Yangzhou, Jiangsu, China; Icahn School of Medicine at Mount Sinai, New York, New York, USA

**Keywords:** TET2, avian leukosis virus subgroup J, Env, autophagic degradation, immunosuppression

## Abstract

Avian leukosis virus subgroup J (ALV-J) poses a significant threat to the poultry industry; yet, our understanding of its replication and pathogenic mechanisms is limited. The Ten-Eleven Translocation 2 (TET2) is an indispensable regulatory factor in active DNA demethylation and immune response regulation. This study reports a significant and time-dependent decrease in TET2 levels following ALV-J infection and shows that the reduction of TET2 protein is mediated by the autophagy pathway. Mechanistically, we found that the accumulation of the Env protein at the late stages of ALV-J infection triggers autophagy, which, in turn, causes the TET2 protein to be exported from the nucleus and subsequently degraded in the cytoplasm. Using CRISPR-Cas9 technology, we generated TET2-deficient chicken macrophages that exhibited increased susceptibility to ALV-J replication, which was reversed by TET2 overexpression. In addition, transcriptome analysis revealed that the absence of TET2 in chicken macrophages impairs the expression of numerous cytokines and innate immune-related genes, particularly those downregulated genes enriched in the RIG-I and Toll-like signaling pathways, leading to enhanced replication of ALV-J *in vitro*. In summary, our research identifies TET2 as an ALV-J restriction factor. However, ALV-J exploits the autophagy machinery to promote the degradation of TET2 protein, thereby disrupting the host’s innate immune responses for viral replication.

**IMPORTANCE:**

ALV-J is a carcinogenic retrovirus that plays a critical role in avian leukosis, primarily affecting chickens. Infection with ALV-J leads to decreased production performance, compromised immune function, and the development of tumors, such as myelocytoma. Currently, there are no effective treatments for ALV-J, making the control of outbreaks a significant challenge with severe economic consequences for the poultry industry. The Env protein of ALV-J has been implicated in the virus’s pathogenicity. Our study shows that ALV-J infection induces autophagy in host cells through its Env protein, leading to the autophagic degradation of TET2, a key epigenetic regulator. The loss of TET2 in macrophages results in the downregulation of innate immune-related gene expression, thereby promoting viral replication. This is the first report to elucidate the role of the ALV-J Env protein in immune suppression *via* TET2 autophagic degradation during ALV-J infection, providing new insights into the mechanisms of viral immune evasion.

## INTRODUCTION

Avian leukosis virus subgroup J (ALV-J) is a retroviral RNA virus that poses a significant threat to chicken flocks, causing subgroup J avian leukosis. This disease leads to reduced production performance, immunosuppression, and tumor development, ultimately resulting in substantial economic losses for the poultry industry (1, 2). Despite its severe impact, effective vaccines or therapeutic strategies against ALV-J have yet to be developed. The ALV-J genome consists of two single-stranded, positive-sense RNA segments that encode three structural proteins: Gag, Pol, and Env. Among these, the Env protein is crucial for ALV-J entry into host cells and serves as a major virulence factor critical for pathogenicity (3–7). However, the complex molecular interactions between the Env protein and host cellular components remain largely unexplored.

Autophagy is a conserved cellular process involving the lysosomal degradation and recycling of cellular organelles and components. This process is not only beneficial for maintaining cellular homeostasis and nutrient recycling but also a key component of the host’s immune response to viral infections. Although autophagy typically safeguards the host by combating pathogens, it is noteworthy that viruses have evolved strategies to either hijack or manipulate this cellular mechanism to their advantage (8, 9). Understanding the interplay between the ALV-J Env protein and autophagy could provide new insights into viral pathogenesis and potential therapeutic targets.

TET2, a member of the ten-eleven translocation (TET) family, plays a crucial role in the continuous oxidation of 5-methylcytosine (5mC) during the process of DNA demethylation (10). Recent research has highlighted the role of TET2 in modulating immune responses and inflammation, suggesting its potential involvement in regulating viral replication within host cells (11, 12). However, aberrant TET2 expression is frequently observed in certain tumor cells and cells infected by viruses (13). For instance, studies have indicated that the stability of the TET2 protein in tumor cells is affected by the autophagy pathway (14). In addition, research has shown that the avian influenza virus (IAV) degrades TET2 mRNA to suppress its expression during infection (15). Similarly, HIV-1 targets the TET2 protein for degradation *via* the ubiquitin-proteasome pathway (16). Therefore, elucidating the expression pattern of the TET2 protein following ALV-J infection is of substantial importance.

In this study, we observed the dynamics of TET2 protein expression in chicken cells post-infection with ALV-J, noting an initial increase followed by a decline. Subsequent studies confirmed that ALV-J markedly reduced TET2 protein levels in these cells during the late stages of infection, utilizing the autophagy pathway. We discovered that the autophagy was initiated by the accumulation of the ALV-J Env protein, which caused TET2 to translocate from the nucleus to the cytoplasm, where it was degraded. Knockout of TET2 demonstrated that its absence led to a weakened antiviral innate immune response, which promoted ALV-J replication. Our findings have shed light on a previously unrecognized mechanism that ALV-J exploits to manipulate the host’s innate immune. Specifically, ALV-J uses its Env protein to initiate autophagy, leading to the degradation of TET2. These results also highlight the pivotal role of the Env protein in the immunosuppressive effects induced by ALV-J infection.

## RESULTS

### Time-dependent suppression of TET2 expression by ALV-J

**Fig 1:**
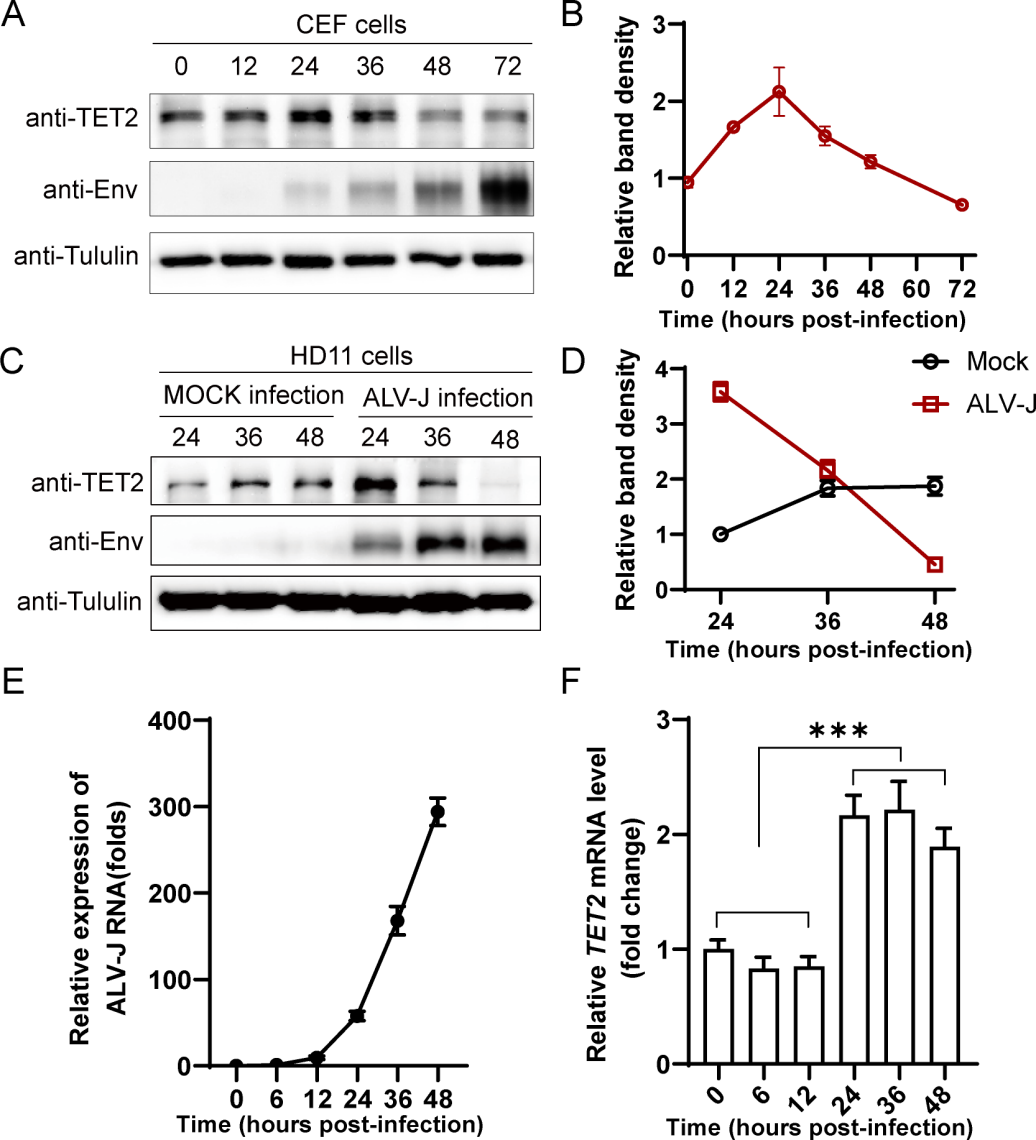
Time-dependent suppression of TET2 expression by ALV-J. (**A**) CEF cells were infected with ALV-J at an MOI of 1 and harvested at 0, 12, 24, 36, 48, and 72 hpi. TET2, viral Env, and Tubulin protein levels were analyzed *via* Western blot. (**B**) Quantification of TET2 to Tubulin protein levels is shown in the graph. (**C**) HD11 cells were infected with ALV-J at an MOI of 1, and samples were collected at 24, 36, and 48 hpi. Protein levels of TET2, viral Env, and Tubulin were examined by Western blot. (**D**) The graph shows the quantification of TET2 to Tubulin protein levels. (**E and F**) HD11 cells were infected with ALV-J at an MOI of 1. The fold changes in ALV-J Env (**E**) and TET2 (**F**) mRNA levels were detected by RT-qPCR at 6, 12, 24, 36, and 48 hpi, using Actin mRNA levels as an internal reference. Error bars represent the mean ± SD of three experiments. Statistical analysis was performed using Student’s t-test. ***P < 0.001.

We examined TET2 expression following ALV-J infection in chicken cells. In CEF cells, TET2 expression initially increased significantly after infection, peaking at 24 h before decreasing at 36, 48, and 72 h (Fig. 1A and B). Similarly, ALV-J infection in HD11 cells showed a significant rise in TET2 protein expression at 24 h post-infection, followed by a decline at 36 and 48 h (Fig. 1C and D). Considering the pivotal role of TET2 in immune regulation, the significant downregulation of TET2 observed in the late stages of ALV-J infection raises our substantial concern for the underlying molecular mechanisms. To investigate further, we examined TET2 mRNA levels in HD11 cells after ALV-J infection (Fig. 1E). The results demonstrated minimal changes in TET2 mRNA expression between 0 and 12 h post-infection. However, a significant increase was observed at 24 h post-infection, and the expression remained stable at 36 and 48 h post-infection (Fig. 1F). These findings suggest that the observed decline in TET2 protein levels is unlikely to result from changes in transcriptional regulation but rather from post-transcriptional regulatory mechanisms.

### ALV-J Env mediates the degradation of TET2 *via* the autophagy-lysosomal pathway

**Fig 2:**
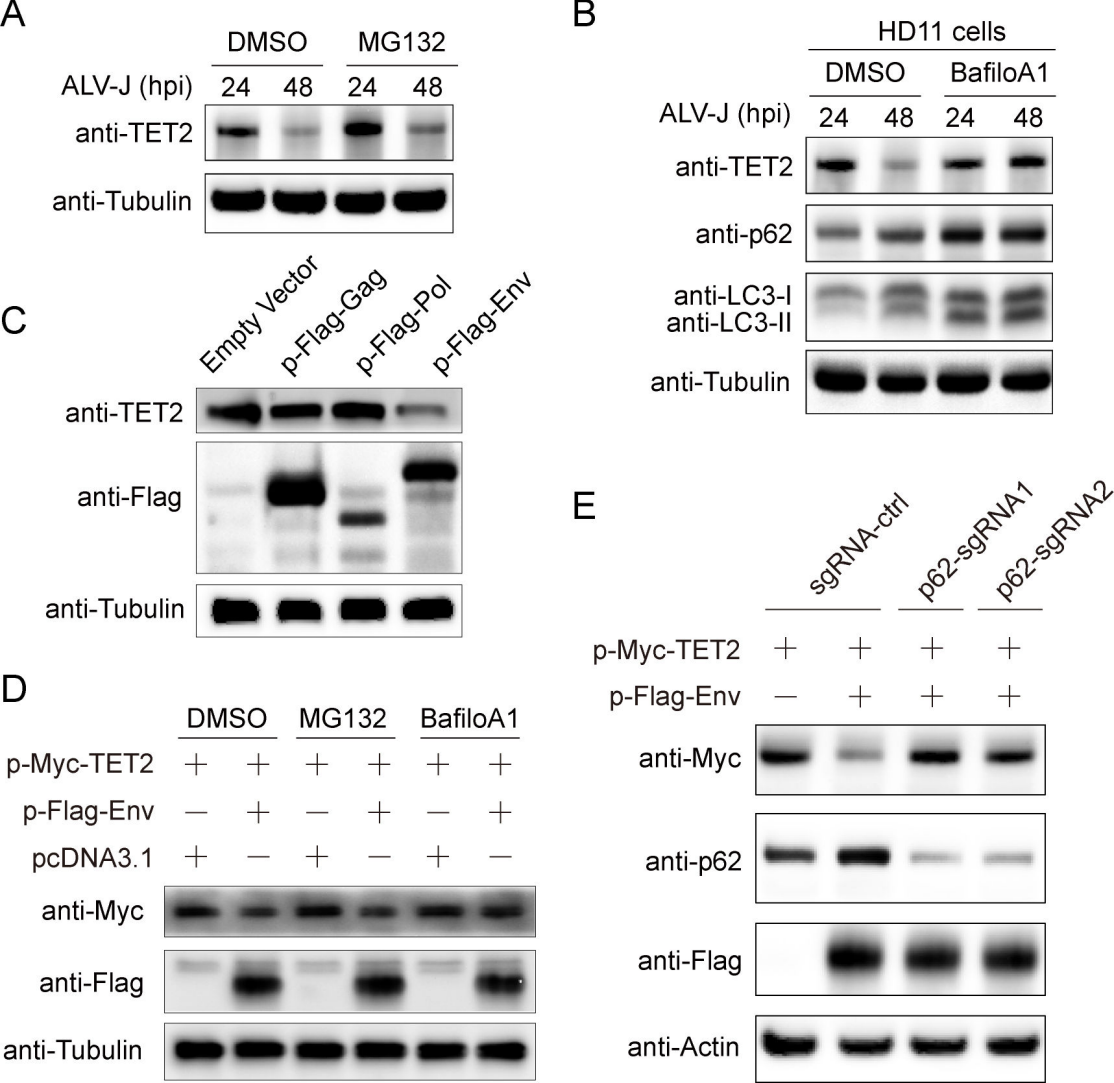
ALV-J Env mediates the degradation of TET2 *via* the autophagy-lysosomal pathway. (**A**) HD11 cells were infected at an MOI of 1 for 24 h and 48 h, then treated with DMSO or MG132 (10 µM) for the last 6 h. Immunoblots were used to detect the expression of TET2, with Tubulin as a loading control. (**B**) HD11 cells were infected at an MOI of 1 for 24 h and 48 h, then treated with DMSO or bafilomycin A1 (BafioA1, 0.5 µM) for the final 4 h. Immunoblots were used to detect the expression of TET2, p62, and LC3B, with Tubulin as a loading control. (**C**) Endogenous TET2 expression levels were detected in HD11 cells transfected with empty vector, Gag, Pol, and Env. (**D**) DF-1 cells were transfected with Myc-TET2 and Flag-Env, and then treated with MG132 (10 µM) or bafilomycin A1 (BafioA1, 0.5 µM) for the last 4 h, and treated with DMSO as control. Cells were harvested for immunoblot analysis to determine TET2 and Env expression, with Tubulin as a loading control. (**E**) DF-1 cells were transfected with p62-targeting sgRNA or control sgRNA vectors, and GFP-positive cells were sorted by flow cytometry. These cells were plated and transfected with Myc-TET2 and Flag-Env for 24 h before harvest for Western blot detection of TET2, Env, and SQSTM1/p62, with Actin as a loading control.

We further elucidated the molecular mechanism of TET2 degradation induced by ALV-J infection through post-translational mechanisms. Prior studies have described two principal pathways of TET2 protein degradation in humans: the ubiquitin-proteasome system and autophagy (14, 16). To examine the relevance of these pathways in ALV-J-induced degradation, HD11 cells were infected and subsequently exposed to specific inhibitors targeting each pathway. Our findings suggest that TET2 degradation is not dependent on the proteasome pathway, as evidenced by the lack of effect of proteasome inhibitor MG132 on TET2 levels (Fig. 2A). By contrast, the autophagy inhibitor bafilomycin A1, which blocks autophagosome maturation, significantly prevented TET2 protein degradation (Fig. 2B). Collectively, these observations strongly suggest that the autophagy pathway plays a crucial role in regulating TET2 degradation.

To investigate whether the autophagy-mediated degradation of TET2 induced by ALV-J is dependent on its viral proteins, overexpression vectors for the three structural proteins of Pol, Gag, and Env were utilized to assess their effects on the levels of endogenous TET2 in HD11 cells. Our results indicated that only the Env protein is the critical determinant for the downregulation of TET2 (Fig. 2C). To further confirm this finding, Myc-TET2 and Flag-Env were co-transfected into DF-1 cells for 24 h, followed by treatment with MG132 or Bafilomycin A1 (Fig. 2D). The results demonstrated that the Env-induced degradation of TET2 was specifically inhibited by Bafilomycin A1, suggesting that the autophagy pathway is a key route for the degradation of TET2 induced by ALV-J Env (Fig. 2D).

Given the role of SQSTM1/p62 as a selective autophagy receptor, it specifically binds to target protein substrates and interacts with the LC3-II protein on the inner membrane of the autophagosome, thereby mediating subsequent lysosomal fusion and substrate degradation. To investigate whether TET2 protein degradation depends on SQSTM1/p62-mediated selective autophagy, we utilized CRISPR-Cas9 to knock down the SQSTM1/p62 protein, effectively inhibiting this pathway. Our results demonstrate that overexpression of Env did not significantly reduce TET2 protein levels in cells lacking SQSTM1/p62. By contrast, a reduction in TET2 protein levels was observed in wild-type DF-1 cells (Fig. 2E). In conclusion, our approaches of pharmacological inhibition and genetic knockdown provide compelling evidence that ALV-J Env downregulates TET2 protein levels through autophagy-related signaling pathways.

### ALV-J Env promotes autophagy and facilitates TET2 autophagic degradation

**Fig 3:**
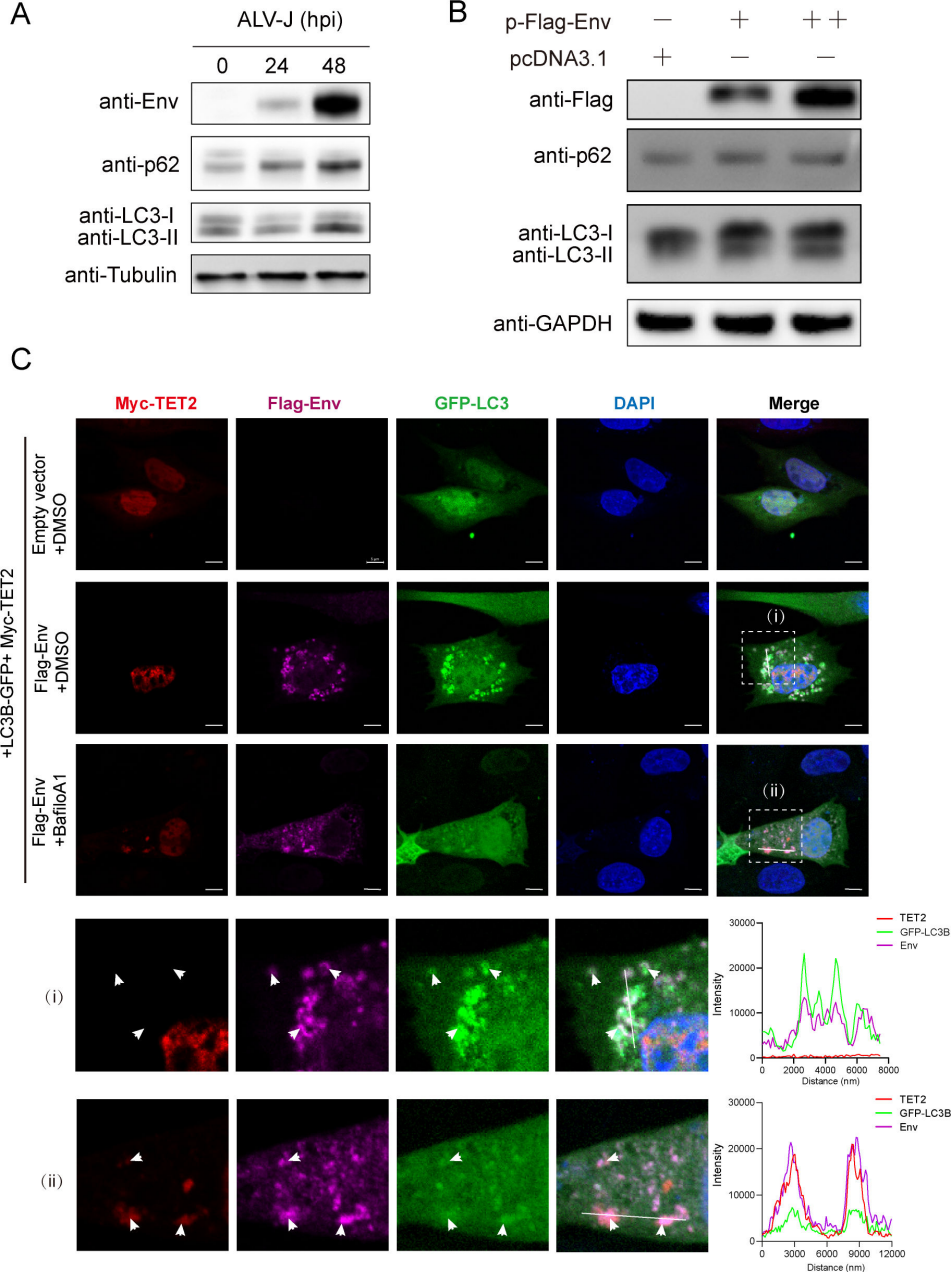
ALV-J Env promotes autophagy and facilitates TET2 autophagic degradation. (**A**) HD11 cells were infected at an MOI of 1 for 24 and 48 h, with uninfected cells as a negative control. Western blot was utilized to assess the levels of Env, SQSTM1/p62, LC3B, and Tubulin as a loading control. (**B**) HD11 cells were transfected with either 1 µg or 2 µg of the Flag-Env expression vector or an empty vector for 36 h. Western blot was utilized to assess the levels of Env, SQSTM1/p62, and LC3B, with GAPDH as a loading control. (**C**) Confocal analysis was performed to assess the colocalization of TET2, Env, and LC3B. DF-1 cells were co-transfected with Myc-TET2 and LC3B-GFP plasmids, or with Myc-TET2, LC3B-GFP, and Flag-Env plasmids, followed by treatment with DMSO or bafilomycin A1 (0.5 µM) for 4 h as indicated, and then subjected to laser confocal analysis. The fluorescence channels for TET2 (excitation/emission at 594 nm), Env(647 nm), GFP, and DAPI were captured, followed by the overlay of images. (**I**) and (ii) represent the typical images of colocalization that were captured, with magnified views of the zoomed areas. The corresponding fluorescence intensity profiles of TET2 (red line), Env (pink line), and LC3B (green line) were obtained using Zeiss ZEN 3.10 analysis software. The scale bar is 5 µm.

To further elucidate the mechanism by which ALV-J or its Env protein triggers the autophagic degradation of TET2, we first investigated the impact of ALV-J infection on different stages of the autophagy pathway. Our findings indicate that the expression of the autophagy marker LC3B-II protein was suppressed at 24 h post-infection but significantly induced at 48 h post-infection (Fig. 3A), suggesting that autophagy is activated at the later stages of infection. Notably, the switch from suppression to induction of autophagy coincides with the dynamic expression of the TET2 protein (Fig. 1A and B). When Env protein was expressed alone, autophagy was also significantly induced, with autophagic activity increasing in correlation with the amount of Env protein (Fig. 3B).

Immunofluorescence analysis showed that, in the absence of Env protein, TET2 was predominantly localized in the nucleus, while LC3B was diffusely distributed in both the nucleus and cytoplasm (Fig. 3C). In contrast, transfection with Env protein-induced punctate aggregation of LC3B, indicative of autophagy activation. When we inhibited lysosomal acidification and protein degradation using Bafilomycin A1, partial translocation of TET2 to the cytoplasm was observed (marked in red), co-localizing with Env and LC3B (Fig. 3C). These findings collectively suggest that Env protein induces autophagy, leading to the translocation of TET2 from the nucleus to the cytoplasm, followed by its autophagic degradation.

### ALV-J Env interacts with TET2 catalytic domain

**Fig 4:**
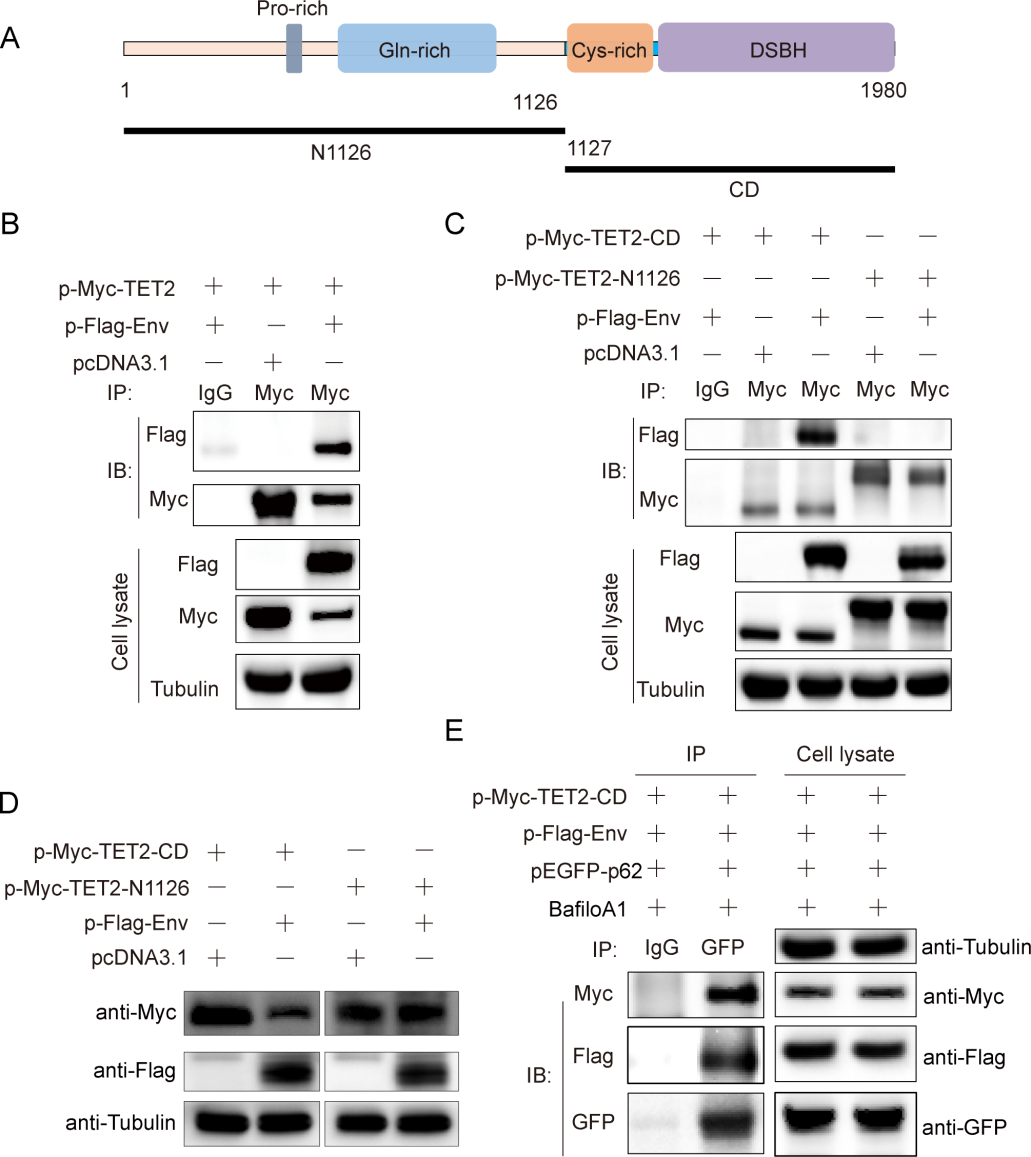
ALV-J Env interacts with TET2 catalytic domain (CD). (**A**) Illustration of the complete structure of chicken TET2 and its truncated variants. (**B**) Interaction between Env protein and TET2. HEK293T cells were transfected with Myc-TET2 alone or co-transfected with Myc-TET2 and Flag-Env to evaluate the interaction between ALV-J Env and TET2. Cell lysates were immunoprecipitated with Myc-tag antibody, followed by Western blot analysis. (**C**) TET2 CD domain interacts with Env. HEK293T cells were co-transfected as indicated for 24 h, and immunoprecipitation with IgG or Myc-tag antibodies and Western blot analysis were conducted on cell lysates. (**D**) Env protein facilitates the degradation of the TET2 CD domain. DF-1 cells were co-transfected as indicated for 24 h and then analyzed by Western blot using cell lysates. (**E**) DF-1 cells were transfected with Myc-TET2-CD, EGFP-SQSTM1/p62, and Flag-Env for 24 h and were treated with bafilomycin A1 (0.5 µM) 4 h prior to cell collection. Immunoprecipitation with a GFP antibody was performed on cell lysates, followed by Western blot analysis.

Confocal microscopy analysis revealed colocalization of Env and TET2 within the cytoplasm, suggesting a potential interaction between these proteins. To further explore this interaction, immunoprecipitation assays were conducted. Following the co-transfection of Env and TET2 into DF-1 cells for 24 h, we confirmed a distinct interaction between TET2 and Env, consistent with our confocal microscopy findings (Fig. 4B). Subsequently, we focused on identifying the specific functional domains of TET2 involved in this interaction. Truncated vectors of TET2 were constructed, including the N-terminal N1126 and the C-terminal catalytic domain (CD), and subjected to IP assays after co-transfection with Env. The results revealed an interaction between TET2-CD and Env, whereas N1126 did not show any interaction with Env (Fig. 4C). Moreover, we investigated whether Env could induce degradation of these truncated domains. Our data indicated that levels of the CD protein decreased upon co-transfection with Env, whereas N1126 remained unaffected, suggesting that Env-mediated degradation of TET2 is dependent on interaction with its catalytic domain (Fig. 4D). Furthermore, we validated the involvement of p62 as a cargo protein in the ALV-J-induced TET2 autophagy-lysosome pathway and explored potential interactions among TET2-CD, Env, and p62. Co-IP results demonstrated interactions among these three components (Fig. 4E). These findings provide insights into the intricate molecular mechanisms underlying the interplay between TET2, Env, and p62 during ALV-J infection.

### TET2 gene knockout alters genome-wide levels of 5-hydroxymethylcytosine

**Fig 5:**
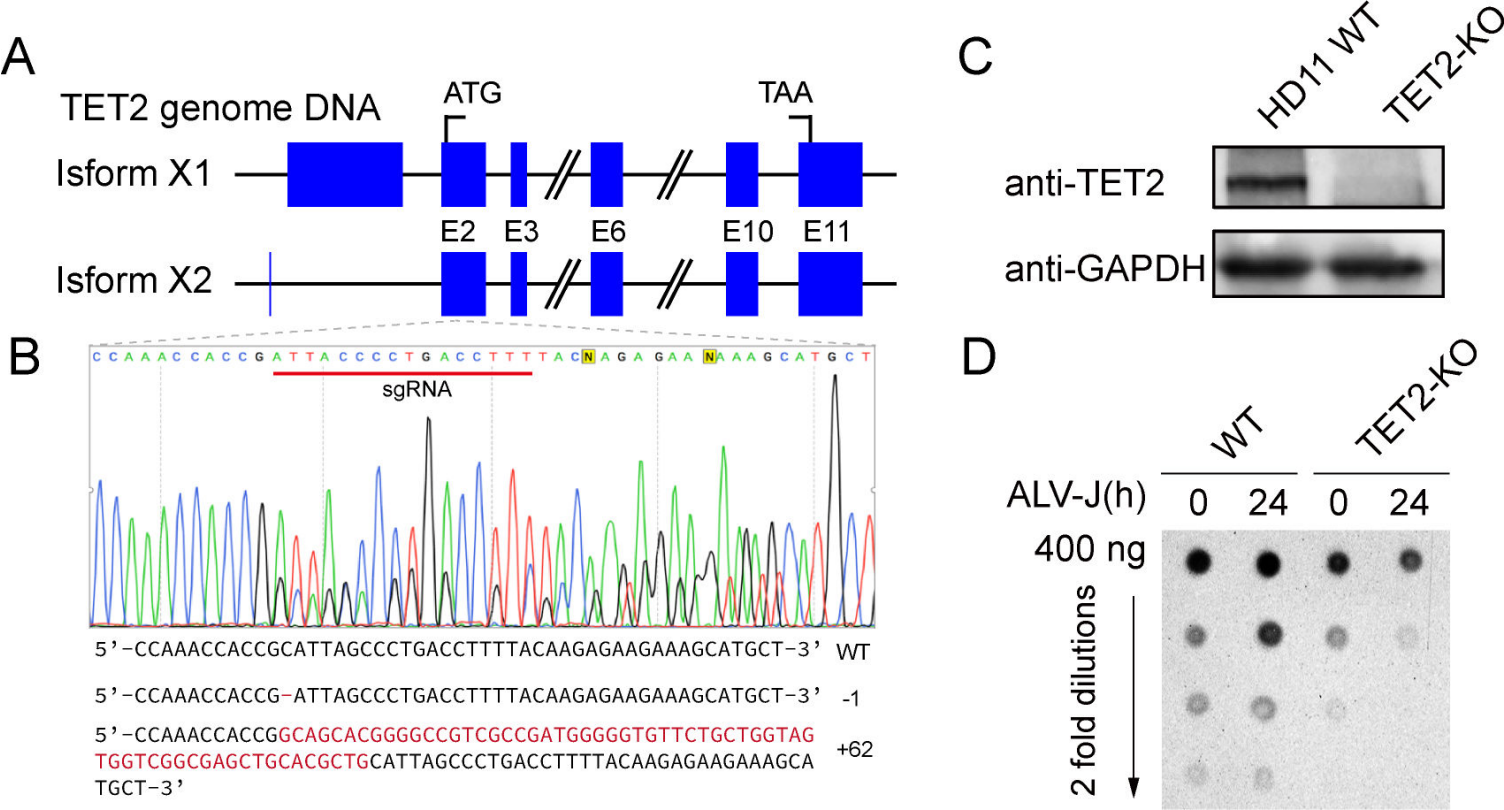
TET2 gene knockout alters genome-wide levels of 5-hydroxymethylcytosine (5hmC). (**A**) A diagram illustrating the chicken TET2 gene locus. (**B**) Genomic sequencing illustrates the sgRNA-directed mutation at the chicken TET2 gene locus. (**C**) Western blot analysis validates the knockout of the TET2 gene in HD11 cells. (**D**) The global level of 5hmC was measured by a dot blot assay. Total genomic DNA was collected from the indicated cells, and the 5hmC levels was determined.

To explore the role of the TET2 gene in HD11 cells and its impact on ALV-J replication, we utilized CRISPR-Cas9 technology to create TET2 knockout HD11 cells. A schematic representation of the chicken TET2 genome is depicted in Fig. 5A. Single-clone TET2 knockout cells was screened, and successful gene editing was confirmed *via* Sanger sequencing (Fig. 5B). Western blot analysis further confirmed the absence of TET2 protein in cells transfected with sgRNA targeting the TET2 gene (Fig. 5C).

Given the critical function of TET2 in regulating genome-wide hydroxymethylation, we evaluated 5-hydroxymethylcytosine (5hmC) levels in both TET2 knockout and wild-type cells before and 24 h after ALV-J infection. Interestingly, we observed a significant increase in global 5hmC levels in wild-type HD11 cells 24 h post-ALV-J infection, consistent with heightened TET2 expression during this period (Fig. 1C). By contrast, TET2 knockout HD11 cells exhibited a substantial decrease in global 5hmC levels at the same time point post-infection. These findings suggest that TET2 plays a crucial role in maintaining 5hmC levels in ALV-J-infected cells, as its knockout disrupted the infection-induced rise in 5hmC (Fig. 5D). This underscores the impact of TET2 knockout on the dynamics of 5hmC expression during ALV-J infection. Further exploration of the molecular mechanisms governing TET2-mediated 5hmC modifications and its effect on gene expression is warranted.

### The expression of TET2 negatively regulates the replication of ALV-J

**Fig 6:**
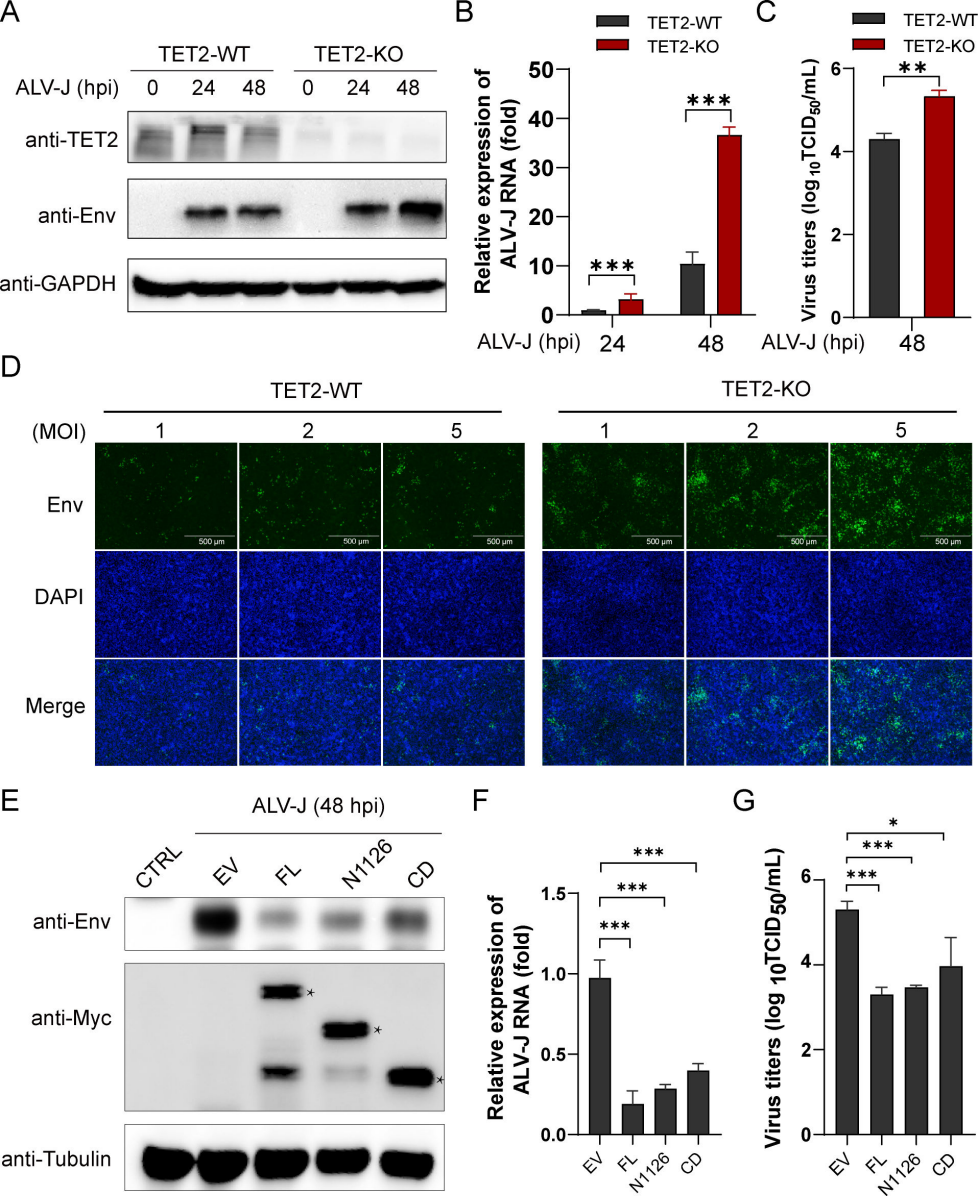
The expression of TET2 negatively regulates the replication of ALV-J. (**A–D**) Deletion of TET2 enhances the replication of ALV-J. (**A**) HD11 cells and TET2-KO cells were infected with ALV-J for 24 and 48 h, respectively, and Western blot analysis was performed to evaluate the expression of TET2 and the Env protein. (**B**) Subsequent to the treatments described in (**A**), qPCR analysis was conducted to measure the levels of ALV-J Env mRNA. (**C**) The viral titer in the supernatant of HD11 and TET2 KO cells at 48 hpi with ALV-J was determined using the TCID_50_ method. (**D**) After infection with indicated viral titers for 48 h, an Indirect immunofluorescence (IFA) assay was used to assess the ALV-J Env protein in HD11 and TET2 KO cells. (**E–G**) Overexpression of TET2 inhibits the replication of ALV-J. (**E**) DF-1 cells were infected with ALV-J (MOI = 1) for 24 h and then transfected with Myc-tagged TET2, deletion mutants Myc-N1126 and Myc-CD, or an empty vector for an additional 24 h. Western blot analysis was conducted to detect the expression of TET2 and ENV proteins. (**F**) Following the treatments described in (**E**), qPCR was performed to detect the expression levels of the ALV-J Env protein. (**G**) After the treatments described in (**E**), cell supernatants were collected and the viral titer was determined using the TCID_50_ method. Data are presented as mean ± SD. A two-tailed Student’s t-test is used for statistical analysis: *P < 0.05, **P < 0.05 and ***P < 0.001.

Next, the impact of TET2 expression on ALV-J replication was evaluated. Initially, the HD11 cells (TET2-WT) and TET2 knockout cells (TET2-KO) were infected with ALV-J, followed by comprehensive analysis *via* western blot, qPCR, TCID_50_, and immunofluorescence assays. Western blot results indicated that at later stages of infection, the expression of ALV-J Env protein was higher in TET2-KO cells compared to TET2-WT cells, suggesting a significant increase in ALV-J replication (Fig. 6A).

This observation was corroborated by qPCR results (Fig. 6B). Furthermore, TCID_50_ assays conducted 48 h post-infection demonstrated substantially elevated virus titers in TET2-KO cells relative to TET2-WT cells (Fig. 6C). Immunofluorescence analysis further validated a notable increase in ALV-J viral particles in TET2-KO cells (Fig. 6D).

The impact of TET2 overexpression on ALV-J replication was subsequently explored. Western blot analysis demonstrated a significant decrease in ALV-J Env protein levels in cells overexpressing TET2 (Fig. 6E). Remarkably, overexpression of both the N-terminal and C-terminal domains of truncated TET2 markedly suppressed ALV-J replication (Fig. 6E). Subsequent qPCR analysis confirmed these observations, showing that overexpression of full-length TET2 and its functional domains significantly inhibited ALV-J replication (Fig. 6F). Consistent with this, TCID_50_ analysis indicated a substantial reduction in virus titers following overexpression of full-length TET2 and its truncated domains (Fig. 6G). These findings suggest that TET2 functions as a negative regulator of ALV-J replication, and this regulatory effect is not entirely dependent of its catalytic domain (CD).

### Immune gene expression impairment by TET2 knockout in HD11 cells

**Fig 7:**
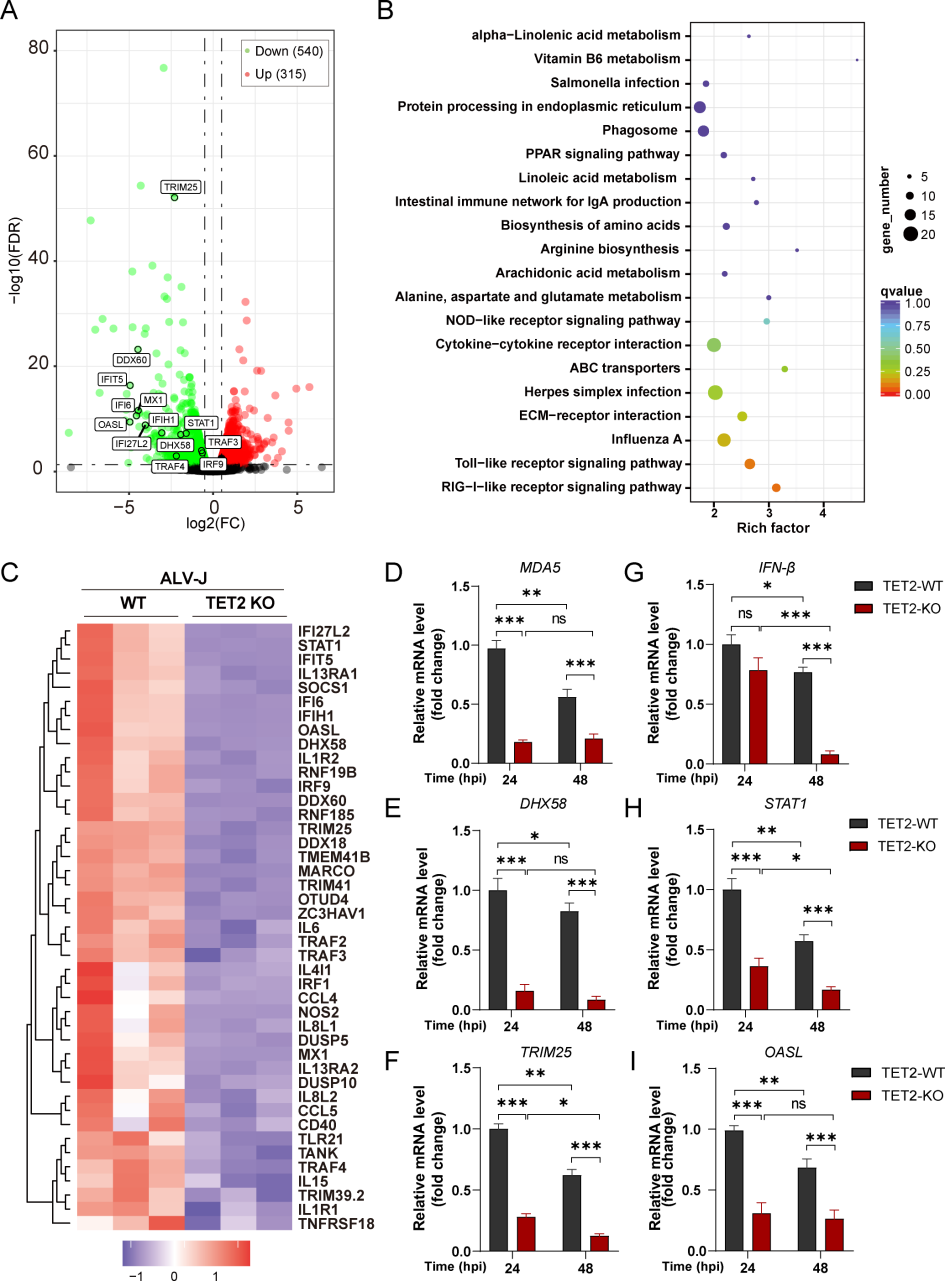
Deletion of TET2 significantly reduces the expression of host innate immune-related genes. (**A**) A volcano plot depicting the genes that are downregulated and upregulated in TET2 knockout HD11 cells as compared to the wild-type cells post 24 h ALV-J infection. (**B**) The genes identified as downregulated in (**A**) are presented according to the KEGG database, with rankings based on Q-values among the top 20 enriched pathways. (**C**) A heatmap illustrating the expression of innate immune-related genes in TET2 knockout HD11 cells as compared to the wild-type cells post 24 h ALV-J infection. (**D–I**) qPCR analysis of the expression changes in innate immune-related genes following TET2 knockout in HD11 cells that were infected for 24 and 48 h. The expression levels for each group were normalized to those of TET2-WT cells infected for 24 h. Data are presented as mean ± SD. A two-tailed Student’s t-test is used for statistical analysis: ns, no significant difference; *P < 0.05, **P < 0.01, ***P < 0.001.

Our research has uncovered that the knockout the TET2 gene in HD11 cells boosts ALV-J replication. To elucidate the underlying molecular mechanisms, we performed transcriptome sequencing analysis. We infected both TET2 knockout and wild-type HD11 cells with ALV-J for 24 h, then collected RNA for transcriptome sequencing and subsequent bioinformatics analysis. The results showed that, compared to wild-type cells, TET2 knockout cells displayed a significant downregulation of numerous genes following ALV-J infection (Fig. 7A; Table S4). Kyoto Encyclopedia of Genes and Genomes (KEGG) enrichment analysis of these differentially expressed genes revealed the RIG-I-like, Toll-like, and influenza A pathways as the top three enriched pathways (Fig. 7B; Table S5), indicating a strong connection between TET2 function and the innate immunity of HD11 cells.

To identify the specific immune genes regulated by TET2, we conducted a heatmap analysis of differentially expressed genes within the significantly enriched pathways (Fig. 7C; Table S6). Our analysis identified two distinct modules: one comprising cytokine genes such as *CCL4*, *IL6*, and *IL1β*, and the other containing type I interferon-related genes like *MDA5*, *MX1*, and *OASL*. KEGG enrichment analysis highlighted the RIG-I-like receptor signaling pathway as significantly enriched following TET2 deletion. We then assessed the transcriptional activity of genes within the RIG-I-like receptor signaling network using qPCR in TET2 knockout and wild-type HD11 cells infected with ALV-J for 24 and 48 h, respectively. We observed that at both 24 hpi and 48 hpi, the expression of key genes in the RIG-I-like receptor signaling cascade was significantly reduced in TET2 knockout cells. These downregulated genes include upstream regulatory factors such as *MDA5*, *DHX58/LGP2*, and *TRIM25* (Fig. 7D through F), as well as downstream effectors like *STAT1* and *OASL* (Fig. 7G through I). Notably, *IFN-β* expression was significantly downregulated only at 48 hpi. These findings suggest that the downregulation of innate immune-related genes in TET2 knockout cells, particularly their impact in the RIG-I-like receptor signaling pathway, could be a key factor in promoting ALV-J replication.

## DISCUSSION

ALV-J, a retrovirus similar to HIV, has developed strategies to effectively evade the host immune defenses and establish its lifecycle within host cells. Macrophages play a crucial role in the innate immune defense against virus and other pathogens, and they are primary targets for ALV-J (17). This virus has been observed to induce apoptosis in macrophages, thereby impairing the host’s immune system (18). Moreover, ALV-J has also been shown to enhance its replication within host cells by disrupting the type I interferon signaling pathway (19, 20). Feng and colleagues noted that the expression of antiviral genes in chicken primary monocyte-derived macrophages infected with ALV-J significantly increased at the early stage of infection but significantly decreased at the later stage (36 hpi), suggesting an immunosuppressive effect of ALV-J (21). However, the molecular mechanisms behind the impaired expression of immune genes by ALV-J infection have not been fully understood. In this study, we have shown that ALV-J infection leads to the consumption of TET2 through post-translational modifications. The absence of TET2 compromises the expression of innate immune genes, weakens the host’s immune defenses, and facilitates the replication of ALV-J. Our findings appear to shed light on why the expression of immune genes is suppressed in the late stages of ALV-J infection.

TET2, a dioxygenase enzyme, catalyzes the conversion of DNA 5mC to 5hmC, thereby regulating gene expression through DNA demethylation (22). Several studies indicate that TET2 is a key factor in maintaining the immune homeostasis of T cells and B cells (23, 24). In this study, we found that ALV-J significantly increased the level of 5hmC within the genome of the infected cells (Fig. 5D). Upon chicken TET2 knockout, there was a significant alteration in the 5hmC dynamics. We found that after TET2 knockout, 5hmC levels decreased following ALV-J infection for 24 h. This indicates that TET2 is essential for the elevation of genomic 5hmC levels, rather than other family members such as TET1 or TET3. On the other hand, the results indicate that the change may be due to the multifunctionality of TET2, as its knockout disrupts the normal dynamics of 5hmC in cells. These results suggest that TET2 may, at least in part, influence the gene expression of ALV-J-infected cells through its mediated demethylation process. Transcriptome sequencing reveals that the TET2 gene knockout in chicken macrophages primarily affects pathways related to innate immunity, such as the RIG-I receptor, Toll-like receptor, and Influenza A signaling pathways (Fig. 7B). Previous reports have indicated that human TET2 is intricately associated with the toll-like receptor pathway, with TET2 playing a pivotal role in initiating the innate immune response in plasmacytoid dendritic cells by being recruited to the TLR7/9 promoter by CXXC5 (25). Recent studies have shown that the degradation of TET2 mRNA by influenza virus can suppresses the expression of critical antiviral immune genes, including ISG20, IFIT5, and ISG15 (15). In our study, transcriptome analysis reveals that the TET2 knockout is significantly associated with the Influenza A signaling pathway (Fig. 7B). Moreover, the expression of the IFIT5 gene is also significantly downregulated in TET2 knockout HD11 cells (Fig. 7A). Notably, for the first time, we have discovered that the knockout of TET2 in chicken macrophages had the most profound effect on the RIG-I-like signaling pathway, significantly reducing the expression of key genes such as *MDA5*, *DHX58/LGP2*, and *TRIM25*. Chickens lack the RIG-I receptors and rely on the MDA5 receptors to recognize RNA viruses (17). Previous studies have indicated that MDA5-mediated type I interferon plays an indispensable role in clearing ALV-J (19, 26, 27). Therefore, our results suggest that the enhanced replication of ALV-J following TET2 knockout may be strongly associated with the interruption of the MDA5-mediated IFNβ signaling pathway.

To ensure their survival within host cells, viruses have developed a variety of strategies, including counteracting or evading host innate immune defenses, which, in turn, enhances their replication. Recent studies have reported the impairment of TET2 expression is a strategy used by viruses to antagonize the antiviral response. For example, the Vpr protein of HIV targets TET2 for degradation *via* the ubiquitin-proteasome pathway, leading to increased IL6 expression, which facilitates HIV replication (16). Similarly, the influenza virus induces TET2 mRNA degradation *via* the PA-X protein, which subsequently impairs the expression of ISG20, IFIT5, and ISG15, thus promoting viral replication (15). In the study, we have demonstrated that the absence of TET2 significantly weakens the innate immune response in host cells, particularly affecting the RIG-I-like receptor pathways, resulting in a significant increase in ALV-J replication during the later stages of infection. Thus, this research reveals a novel example of a virus interfering with TET2 expression, suggesting that the disruption of TET2 may be a widespread strategy employed by viruses to facilitate their replication.

Autophagy is an essential intracellular degradation pathway that begins with the formation of autophagosomes, which then fuse with lysosomes to degrade enclosed cytoplasmic materials. The process is divided into two types: non-selective and selective autophagy (28). Non-selective autophagy degrades substrates without specificity, whereas selective autophagy involves receptors such as p62/SQSTM1 binding to specific substrates and associating with LC3 (Atg8 in yeast and plants) to target them for degradation within autophagosomes (29). Viruses are known to exploit this process to evade the host immune response and enhance their replication, with a variety of dynamic interactions observed between selective autophagy and different viruses (9, 30). For instance, HIV can employ distinct strategies during its life cycle, either inhibiting autophagy or leveraging it for replication, depending on the stage of infection (31, 32). Prior studies have indicated that ALV-J suppresses autophagy to induce apoptosis in the DF-1 chicken embryo fibroblast cell line during the early stages of infection (33, 34). Our findings further elucidate the complex interplay between ALV-J and autophagy, particularly involving the process of nuclear autophagy of TET2. Nuclear autophagy is a conserved mechanism that involves the selection and transfer of nuclear components to the cytoplasm (35). In cancer cells, p53 has been demonstrated to facilitate the translocation of TET2 from the nucleus to cytoplasmic autophagosomes, leading to its degradation (14). On the other hand, research by Dou and colleagues has highlighted that LC3 shifts from the nucleus to the cytoplasm and is essential for the nuclear-cytoplasmic translocation process required for cellular autophagy. LC3 binds to the nuclear protein laminB1, promoting its translocation from the nucleus to the cytoplasm (36). Furthermore, Xu et al. discovered that the nuclear protein SIRT1 is transported to the cytoplasm by the autophagy-associated protein LC3 during senescence and is degraded by the cytoplasmic autophagosome-lysosome system (37). In our study, we illustrate that ALV-J Env promotes the degradation of TET2 through nuclear autophagy, with observed co-localization of ALV-J Env, TET2, and LC3B in the cytoplasm (Fig. 3). From the confocal microscopy results, we observed that LC3B is evenly distributed between the nucleus and the cytoplasm in cells not expressing Env (Fig. 3). However, when Env is expressed, it induces autophagy, resulting in a significant increase in LC3B distribution within the cytoplasm (Fig. 3). Our findings are consistent with previous reports (36). Nevertheless, further evidence is required to demonstrate that LC3B is involved in the nucleocytoplasmic translocation of TET2. In addition, this study highlights the impact of TET2 deficiency on innate immune signaling pathways but does not further elucidate how TET2 regulates the expression of immune genes.

**Fig 8:**
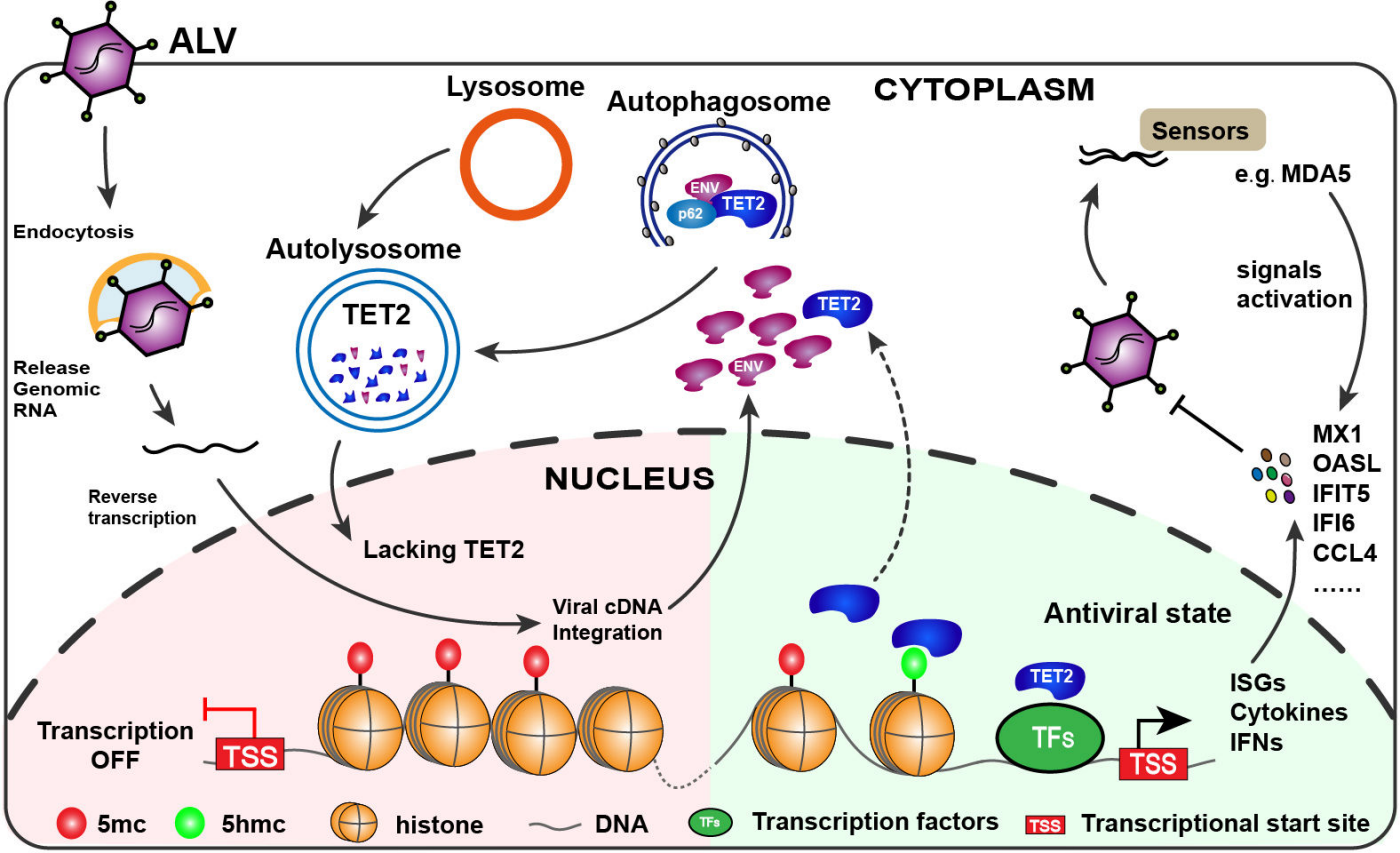
Proposed model depicting the evasion of host immune defenses by ALV-J through the autophagic degradation of TET2. TET2 is essential for maintaining the expression of a series of cytokines and immune-related genes that are key to inhibiting the proliferation of ALV-J. Conversely, the Env protein of ALV-J induces autophagy, which facilitates the translocation of TET2 from the nucleus to the cytoplasm. In the cytoplasm, the interaction between the Env protein and TET2 leads to the selective autophagic degradation of TET2, preventing it from effectively activating the innate immune response and thus promoting viral replication.

In conclusion, our current research demonstrates that TET2 plays a positive role in regulating innate immune signaling by sustaining the expression of a variety of cytokines and immune-related genes, effectively acting as a defense barrier. Notably, to overcome this barrier, ALV-J triggers autophagy *via* its Env protein, which facilitates the translocation of TET2 from the nucleus to the cytoplasm, where it is then degraded through a p62/SQSTM1-dependent autophagy process (Fig. 8). These findings deepen our comprehension of how ALV-J induces autophagy to cause immune suppression and promote its proliferation, offering new perspectives on the interaction between TET2 and ALV-J that could inform the development of antiviral therapies.

## MATERIALS AND METHODS

### Cells and virus

The DF-1 chicken fibroblast cell line (ATCC no. CRL-12203) and the HEK 293T cell line (ATCC no. CRL-3216) were obtained from the American Type Culture Collection (ATCC). Professor Xin'an Jiao of Yangzhou University kindly provided the HD11 cell line, a chicken macrophage-like cell line. Primary chicken embryonic fibroblasts (CEFs) were derived from specific-pathogen-free (SPF) White Leghorn chicken embryos at 10 days of age, following established protocols (38). All cell lines were maintained at 37°C in a CO2 incubator in Dulbecco’s Modified Eagle Medium (DMEM) supplemented with 10% fetal bovine serum (FBS) and antibiotics (100 U/mL penicillin and 100 µg/mL streptomycin).

The ALV-J strain JS09GY3 utilized in the experiments has been previously reported (27). For virus amplification, the DF-1 cell line was employed in accordance with standard protocols. Briefly, after a 2 h incubation with ALV-J, the supernatant was removed. The cells were then washed twice with PBS and supplemented with fresh medium containing 1% FBS. Following this, the cells were incubated at 37°C for 7 days or longer. The supernatant was collected and stored at −80°C for use in subsequent infection assays.

### Plasmid constructs and transfection

The cDNA sequences that encode for chicken TET2 or SQSTM1/p62 were amplified from HD11 cells and then subcloned into the vectors pCAGGS-Myc or pEGFP, respectively. Truncated variants of TET2 were created through PCR amplification, using the wild-type protein construct as a template. Each plasmid construct was validated by Sanger sequencing. The PCR primer sequences for the construction of the expression vectors are listed in Table S1. GFP-LC3 was sourced from Miaoling Co. Ltd. HD11 cells were treated with either bafilomycin A1 or MG132 for 4 h prior to collection. For the transfection experiments, DF-1 or 293T cells were transfected with the appropriate plasmids as illustrated in the figures, using QuickShuttle transfection reagent (Biodragon) in accordance with the manufacturer’s guidelines.

### qRT-PCR

After removing the cell supernatant, cells were washed with chilled PBS and RNA was extracted using TRIzol reagent (Invitrogen) according to the manufacturer’s protocol. One microgram of RNA was reverse transcribed into cDNA with the HiScript SuPerMIX for qPCR (+gDNA wiper) kit from Vazyme. Real-time PCR was executed with the 2 × ChamQ Universal SYBR qPCR Master Mix (Vazyme) on a BioRad CFX Connect Real-time PCR detection system. Relative expression levels were normalized to reference genes, *β-Actin* or *GAPDH*, using the comparative Ct (2^−∆∆Ct^) method. The qPCR primers used in this study are listed in Table S2.

### Western blot and antibodies

Western blot was performed as previously described (38). RAPA cell lysis buffer and Protease/Phosphatase inhibitor cocktail were procured from Cell Signaling Technology. Protein A/G magnetic beads were obtained from Thermo Scientific. MG-132 and Bafilomycin A1 were also sourced from MedChemExpress. Primary antibodies against Tubulin, GAPDH, DYKDDDDK-tagged FLAG, and Myc-tag were acquired from MBL. Antibodies against TET2 (#18950), SQSTM1/p62 (#5114), and LC3A/B (#2741) were obtained from Cell Signaling Technology. Anti-5hmC antibody, Goat anti-Mouse IgG horseradish peroxidase (HRP) conjugate (ab6789), and Goat anti-Rabbit IgG HRP conjugate (ab6721-1001) were sourced from Abcam. CoraLite 647-conjugated AffiniPure F(ab′)2 Fragment Donkey Anti-Rabbit IgG (SA00014-7) for fluorescent detection was purchased from Proteintech. ABflo594-conjugated Goat Anti-Rabbit IgG (AS039), Rabbit polyclonal antibody (pAb) Control IgG (AC005), and Mouse Control IgG (AC011) for use as isotype controls in immunoassays were sourced from ABclonal.

### CRISPR/Cas9 genomic editing

For SQSTM1/p62 or TET2 knockout, we followed our established method (39). Briefly, single-guide RNAs (sgRNAs) targeting the chicken SQSTM1/p62 or TET2 gene were designed using the CHOPCHOP tool (http://chopchop.cbu.uib.no/). Each sgRNA was co-transfected into cells along with the Cas9-T2A-GFP expression vector. Following a 48 h incubation period, GFP-expressing cells were isolated using fluorescence-activated cell sorting (FACS) and subjected to limiting dilution in a 96-well plate to establish single-cell-derived clones. DNA sequencing and western blot analysis were performed, verifying the successful knockout cell. The primers used to generate the SQSTM1/p62 or TET2 sgRNAs vector are listed in Table S3.

### DNA extraction and dot blot analysis

Dot blot analysis was performed in accordance with our prior research (39). In brief, the DNA was isolated using the FastPure Cell/Tissue DNA Isolation Mini Kit from Vazyme. Each sample was prepared with 400 ng of DNA for a series of gradient dilutions. The diluted DNA samples were spotted onto a positively charged nylon membrane and cross-linked using ultraviolet light for 30 min. The membrane was then blocked to prevent non-specific binding and incubated overnight with a primary antibody specific for 5hmC. Finally, the membrane was subjected to imaging analysis to determine the levels of 5hmC.

### RNA-seq analysis

Following a 24 h ALV-J infection period, HD11 and TET2-KO cells were collected for total RNA extraction. Subsequently, three biological replicates of RNA samples for each treatment group were sent to BioMarker Biotech (Beijing, China) for transcriptome library preparation and subsequent bioinformatics analysis. For RNA-seq library preparation, 1 µg of RNA per sample was used, following the manufacturer’s (NEB, USA) instructions for the NEBNext Ultra RNA Library Prep Kit for Illumina. RNA sequencing was performed on the Illumina HiSeq2000 platform. High-quality reads from sequencing were aligned to the chicken genome (GGA5.0) using TopHat v2.0.9. Expression levels of each gene were normalized to fragments per kilobase of transcript per million mapped reads (FPKM). Cuffdiff was then used to compare mRNA levels among the samples. Significantly differentially expressed genes were identified using a false discovery rate (FDR) significance threshold of <0.05 and a fold change of ≥2.0. Finally, the generation of volcano plots, heatmaps, and KEGG pathway analyses was performed using open-source R programming (version 3.6.1).

### Co-immunoprecipitation

Cell lysates were prepared by incubating cells in NP40 buffer with 150 mM NaCl, 1 mM EDTA, 1% Nonidet P-40, and a protease and phosphatase inhibitor cocktail for 30 minutes. The lysate was then centrifuged at 14,000 × *g* in a pre-chilled centrifuge to obtain the supernatant. This supernatant was incubated with the appropriate IgG or antibodies at 4°C for 4 h with rotation. Protein A/G magnetic beads were added, followed by a 2 h incubation at room temperature. The mixture was applied to a magnetic rack to separate bead-bound proteins and then washed three times with pre-chilled NP40 buffer. Elution of the magnetic beads was performed with a 2× loading buffer. The eluted proteins were subjected to SDS-PAGE, followed by immunoblot analysis as shown in the figure.

### Confocal fluorescence microscopy

DF-1 cells were plated onto coverslips in 24-well plates. The following day, these cells were transfected with the specified plasmids, and after 24 h, they were treated with or without bafilomycin A1 for another 4 h. Subsequently, the cells were fixed at room temperature for 10 minutes with a fresh 4% paraformaldehyde solution in PBS, permeabilized for 10 minutes with 0.2% Triton X-100 in PBS, and blocked for 30 minutes in PBS containing 5% donkey serum albumin (DSA). After washing, the cells were incubated at 4°C overnight with Myc-tag and Flag-tag antibodies. On the next day, the cells were washed three times with pre-chilled PBS, then stained at room temperature with CoraLite 647-conjugated AffiniPure F(ab′)2 Fragment Donkey Anti-Rabbit IgG (Proteintech) and ABflo555-conjugated Goat Anti-Rabbit IgG (Abconal). One hour later, the cells were stained with DAPI for 5 minutes for nuclear visualization. The coverslips were subsequently washed with PBS and then mounted using SlowFade Gold antifade mountant (ThermoFisher). Finally, images were captured using a Zeiss LSM 810 confocal microscope and analyzed with Zeiss software Vision 3.8 (Zeiss).

### Statistical analysis

The statistical significance between the experimental groups was assessed using a Student’s t-test, conducted with GraphPad Prism 5.0 software. The data were presented as means ± standard deviation (SD). *P* < 0.05 was considered statistically significant.

## Data Availability

The data sets generated for this study have been deposited to the NCBI Sequence Read Archive (SRA) under BioProject accession number PRJNA1203918.

## SUPPLEMENTAL MATERIAL

Table S1 jvi.02003-24-s0001.xlsx Primers used for constructing the vector in this study.

Table S2 jvi.02003-24-s0002.xlsx qPCR primers used in this study.

Table S3 jvi.02003-24-s0003.xlsx Synthesized sgRNAs that target chicken TET2 or SQSTM1/p62 gene.

Table S4 jvi.02003-24-s0004.xlsx Total differentially expressed genes between TET2-WT and TET2-KO HD11 cells infected with ALV-J.

Table S5 jvi.02003-24-s0005.xlsx KEGG pathway enrichment analysis of down-regulated differentially expressed genes.

Table S6 jvi.02003-24-s0006.xlsx Differentially expressed genes are enriched in the antiviral innate immune pathway.

ASM does not own the copyrights to Supplemental Material that may be linked to, or accessed through, an article. The authors have granted ASM a non-exclusive, world-wide license to publish the Supplemental Material files. Please contact the corresponding author directly for reuse.
